# Crystal structure of (*Z*)-2-fluoro­benzyl 2-(5-fluoro-2-oxoindolin-3-yl­idene)hydrazinecarbodi­thio­ate dimethyl sulfoxide monosolvate

**DOI:** 10.1107/S205698902600561X

**Published:** 2026-06-02

**Authors:** Mohd Abdul Fatah Abdul Manan, David B. Cordes, Aidan P. McKay

**Affiliations:** a Faculty of Applied Sciences, Universiti Teknologi MARA, 40450 Shah Alam, Selangor, Malaysia; b EaStCHEM School of Chemistry, University of St Andrews, St Andrews, Fife, KY16 9ST, United Kingdom; University of Aberdeen, United Kingdom

**Keywords:** crystal structure, fluorine, halogen bonding, hydrogen bonding, chalcogen bonding

## Abstract

The title solvate, C_16_H_11_F_2_N_3_OS_2_·C_2_H_6_OS, crystallized with two independent mol­ecules (*A* and *B*) in the asymmetric unit. Both mol­ecules exhibit an L-shaped geometry but slightly differ in the orientation of the *o*-fluoro­benzyl ring with respect to the 5-fluoro­isatin ring: this dihedral angle is 89.1 (5)° in mol­ecule *A* and 86.3 (5)° in mol­ecule *B*. In the crystal, the *A* and *B* mol­ecular conformations are stabilized *via* N—H⋯O intra­molecular hydrogen bonds. Both independent mol­ecules are linked *via* a weak directional C—H⋯F inter­molecular hydrogen bond, resulting in the formation of dimers. The supra­molecular network mainly comprises C_ar_—H⋯F and C_ar_—H⋯S hydrogen bonds, homohalogen Type I F⋯F halogen⋯halogen bonds and S⋯O chalcogen bonds.

## Abstract 2

The crystal structure of the title solvate features homohalogen (F⋯F) Type I inter­molecular inter­actions, non classical C_ar_—H⋯F and C_ar_—H⋯S hydrogen bonds, as well as S⋯O chalcogen bonds.

## 1. Chemical context

Isatin-derived imines have been explored extensively in organic and coordination chemistry owing to their versatile coordination behavior and significant role in solid state-organization supra­molecular chemistry (Shanmugam *et al.*, 2025 ▸; Pokharel *et al.*, 2025 ▸; Shahi *et al.*, 2023 ▸). In particular, isatin-based fluorinated di­thio­carbazate imines incorporate both thio­amide and azomethine functionalities as well as fluorine frameworks, providing multiple hydrogen-bond donors and acceptors, which enables fine-tuning of supra­molecular architecture in solid state, thereby altering the physical and chemical properties of materials (McKay *et al.*, 2025 ▸; Abdul Manan *et al.*, 2024 ▸). Intermolecular interactions involving fluorine play an important role in modulating crystal packing and mol­ecular assembly (Singla *et al.*, 2023 ▸; Sakshi *et al.*, 2025 ▸; Das *et al.*, 2026 ▸). Various fluorine-mediated inter­actions in chalcogen-containing compounds are capable of engaging multiple inter­molecular inter­actions in the crystal network that cooperatively stabilize the crystal structures (McKay *et al.*, 2026 ▸; Pessoa *et al.*, 2025 ▸; Dey *et al.*, 2021 ▸). For example, our recent crystallographic study on 4-fluoro­benzyl (*Z*)-2-(2-oxoindolin-3-yl­idene)hydrazine-1-carbodi­thio­ate highlighted the role of aromatic organic fluorine in stabilizing the crystal packing by forming dimers through the C—H⋯F—C supra­molecular synthon (Abdul Manan *et al.*, 2024 ▸). In this perspective, the present work reports on synthesis and crystal structure of the title compound, with particular emphasis on the role of fluorine in the supra­molecular assembly.
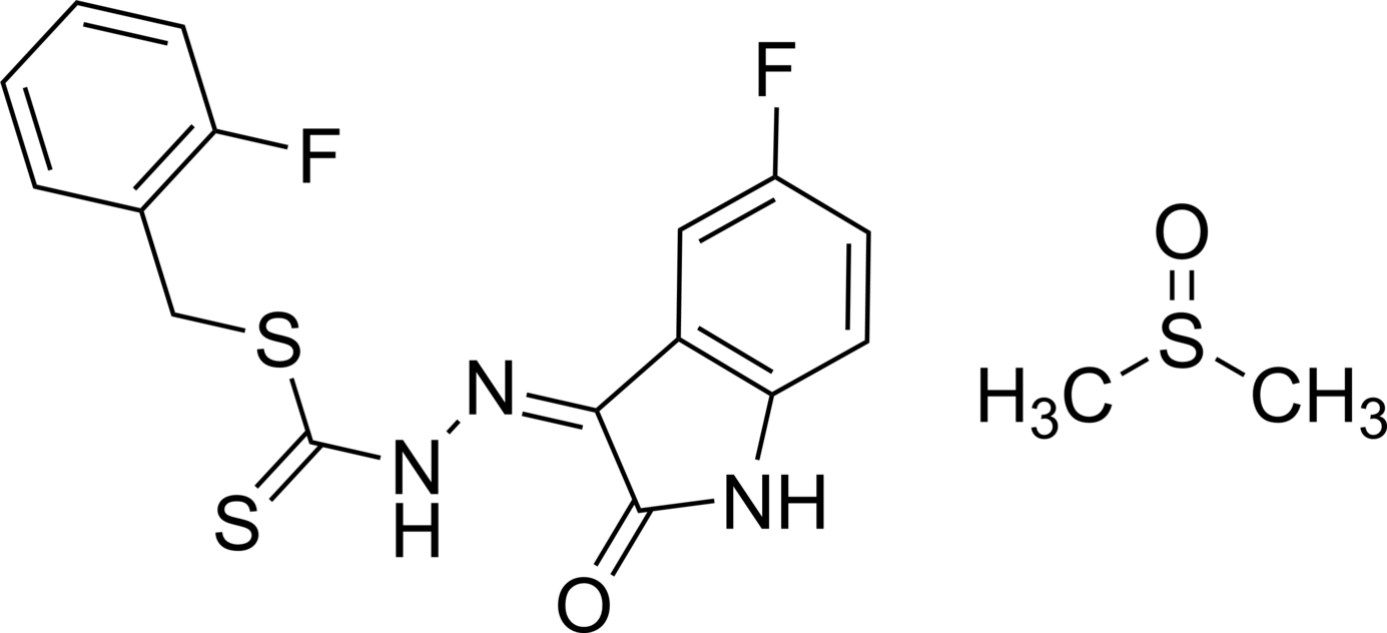


## 2. Structural commentary

**Figure 1:**
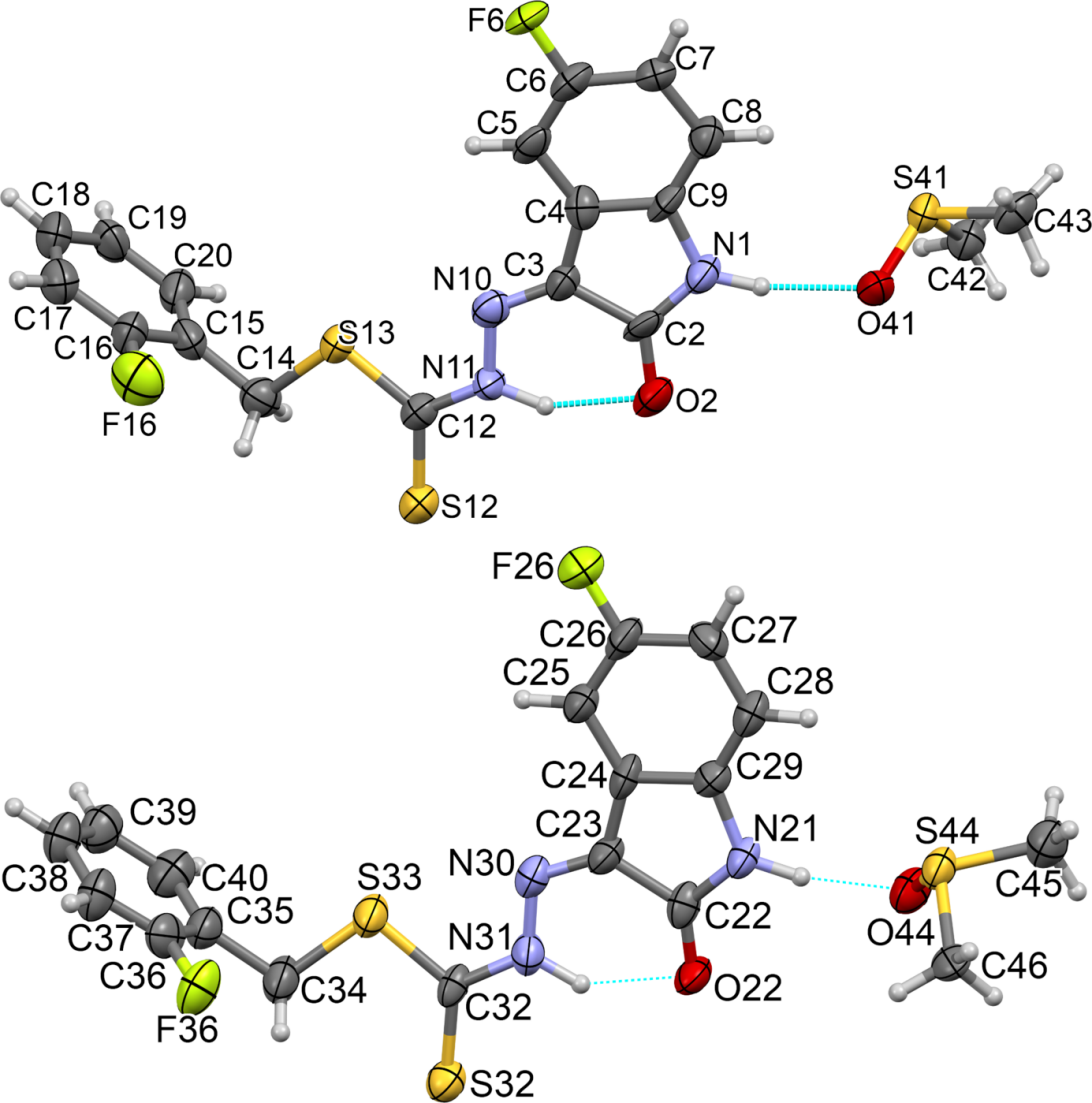
The mol­ecular structure of mol­ecules *A* (top) and *B* (bottom) of the title compound with displacement ellipsoids drawn at the 50% probability level. Hydrogen bonds are shown as blue dashed lines.

The title compound crystallized with two independent mol­ecules (*A* and *B*) in the asymmetric unit (Fig. 1 ▸), each solvated by a mol­ecule of DMSO. The mol­ecular structure comprises one ortho fluoro substituted benzyl ring, a rigid and planar methyl­idenehydrazinecarbodi­thio­ate moiety and a 5-fluoro­isatin ring. The bond lengths and bond angles are within the normal ranges and are consistent with those reported for analogous compounds (McKay *et al.*, 2025 ▸, 2026 ▸). Both mol­ecules adopt an L-shaped geometry with slight conformational differences, particularly in the orientation of the *o*-flurobenzyl ring with respect to the 5-fluoro­isatin group, as reflected in the dihedral angles of 89.1 (5)° in mol­ecule *A* and 86.3 (5)° in mol­ecule *B*. The N—H hydrazine fragment forms an intra­molecular hydrogen bond with the carbonyl oxygen atom of the γ lactam, generating an *S*(6) motif that stabilizes and effectively locks the C=N azomethine bond in the *Z* configuration.

## 3. Supra­molecular features

**Figure 2:**
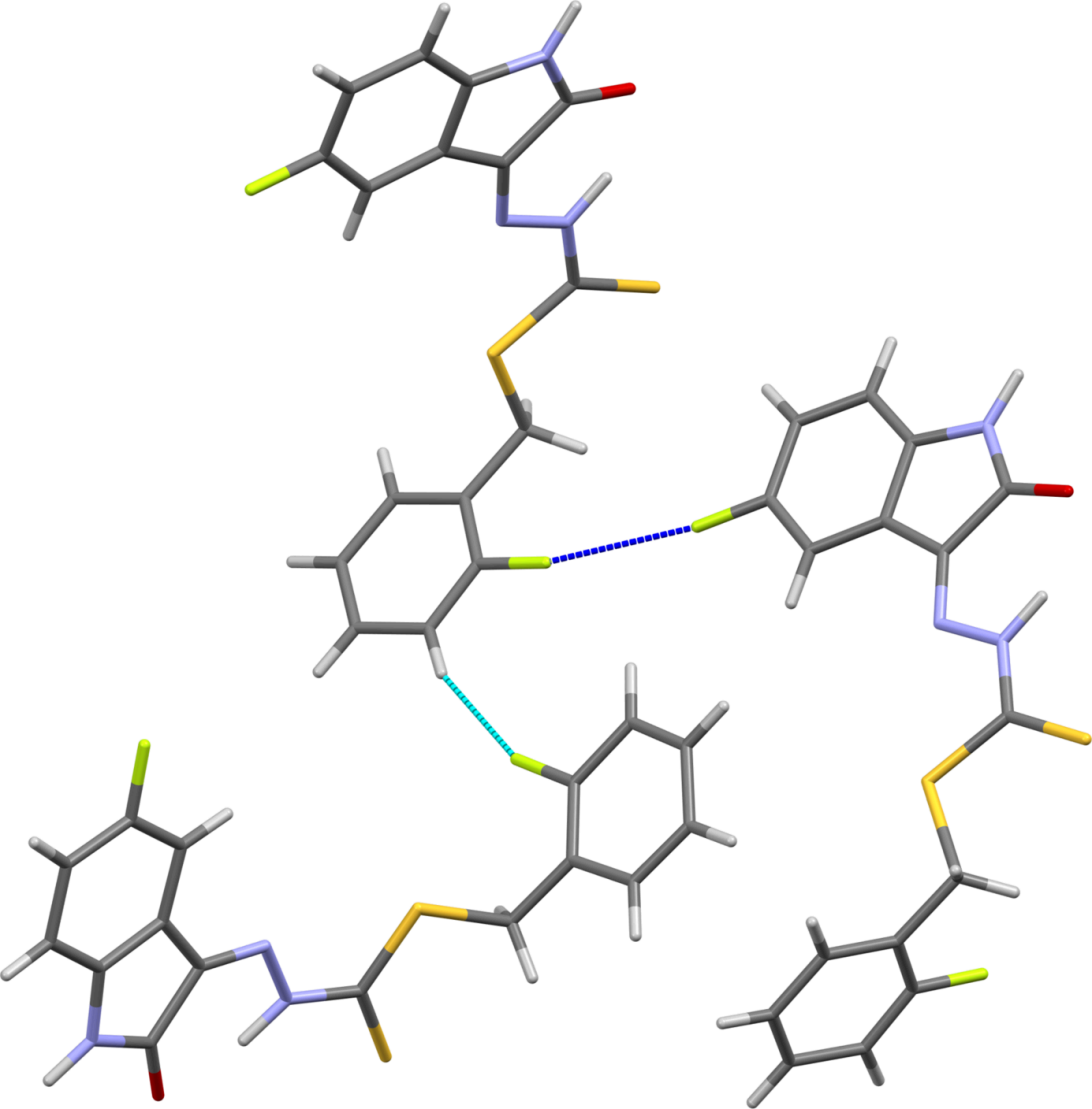
View of adjacent mol­ecules inter­acting *via* C_ar_—H⋯F hydrogen bonds (blue dashed lines) and F⋯F halogen bonds (dark-blue dashed lines).

Details of the hydrogen bonding are summarized in Table 1 ▸. Each mol­ecule inter­acts with its corresponding DMSO solvent mol­ecule *via* N–H⋯O inter­molecular hydrogen bonding from the N–H of the 5-fluoro­isatin ring, resulting in the formation of discrete mol­ecule–solvate pairs. In the crystal, the *A* and *B* mol­ecules are linked in a head-to-head manner *via* a non-classical inter­molecular C_ar_—H⋯F hydrogen bond with an H⋯F distance of 2.27 Å and C—F⋯H angle of 160°, resulting in the formation of dimers. Alongside this hydrogen bond, there is a homohalogen F⋯F halogen⋯halogen bond between the fluoro­benzyl fluorine not taking part in the C_ar_—H⋯F hydrogen bond and the fluorine of an adjacent fluoro­isatin of a symmetry-related mol­ecule (Fig. 2 ▸) [F26⋯F36 = 2.63 (13) Å, C26—F26⋯F36 = 169.5 (10)°, C36—F36⋯F26 = 157.4 (11)°], which adopts a Type I geometry (Δθ = 12.1°, where Δθ = |θ_1_ – θ_2_|; Tothadi *et al.*, 2013 ▸). These inter­actions involving fluorine give rise to two-dimensional sheets in the (001) plane, and when considered together with other non-classical C_ar_—H⋯S and C_ar_—H⋯O hydrogen and chalcogen bonds involving the DMSO solvates, these inter­actions consolidate the crystal packing into a three-dimensional network and are comparable to those found in the bromo and chloro counterparts, indicating preservation of the principal supra­molecular inter­actions (McKay *et al.*, 2025 ▸, 2026 ▸).

## 4. Database survey

A search of the Cambridge Structural Database (CSD version 6.01, updated February 2026; Groom *et al.*, 2016 ▸) for 2-benzyl-2-(2-oxoindolin-3-yl­idene)hydrazinecarbodi­thio­ate with any substituents returned eleven matches. These include the unsubstituted compound and a solvate (EPOFAR, EPOFEV; Ali *et al.*, 2011 ▸), compounds with fluoro, chloro, bromo (ABOROA, ABOSAN, ABORUG; Abdul Manan *et al.*, 2011 ▸), nitro (JASGUJ; Pereira *et al.*, 2021 ▸) and methyl-substituted isatin groups (Abdul Manan *et al.*, 2023 ▸), as well as two compounds with differing fluorination positions on the benzyl group (FOLXIR; Abdul Manan *et al.* 2024 ▸; OSEWES; McKay *et al.*, 2026 ▸), and also two compounds with substituents on both the isatin and benzyl groups (EMALOX; McKay *et al.*, 2025 ▸; OSEWIW; McKay *et al.*, 2026 ▸). All of these compounds show the same geometry as the title compound with the isatin and methyl­enehydrazinecarbodi­thio­ate groups approximately coplanar and the terminal phenyl oriented approximately orthogonal to this.

## 5. Synthesis and crystallization

The 2-fluoro­benzyl hydrazinecarbodi­thio­ate precursor was synthesized using our previously published methods (McKay *et al.*, 2025 ▸). A solution of 5-fluoro­isatin (1.65 g, 10.0 mmol, 1.0 e.q) in hot ethanol (50 ml) was added to a solution of 2-fluoro­benzyl hydrazinecarbodi­thio­ate (2.16 g, 10.0 mmol, 1.0 e.q) in hot ethanol (50 ml). The mixture was heated (353 K) with continuous stirring for 15 min then allowed to cool to room temperature and stand for about 20 min, until a precipitate formed, which was collected by filtration and dried over silica gel. The crude solids were purified by recrystallization from ethanol solution to yield a yellow solid (yield: 3.09 g, 85%). m.p 501–502 K. FT–IR (KBr, ν, cm^−1^): 3222 (NH), 1690 (C=O), 1632 (C=N), 1076 (C=S), 1148 (N—N). ^1^H NMR (400 MHz, DMSO-*d_6_*) δ: (p.p.m): 4.56 (*s*, 2H), 6.94 (*dd*, *J* = 8.6, 4.2 Hz, 1H), 7.18–7.28 (*m*, 3H), 7.34-7.41 (*m*, 2H), 7.57 (*td*, *J* = 7.68, 1.75 Hz, 1H), 11.37 (*s*, 1H), 13.96 (*s*, 1H). Crystals suitable for X-ray diffraction were grown by slow evaporation of a di­methyl sulfoxide solution at room temperature.

## 6. Refinement

Crystal data, data collection and structure refinement details are summarized in Table 2 ▸. The N-bound H atoms were located in a difference Fourier map and refined isotropically subject to a distance restraint, and with *U*_iso_(H) = 1.2*U*_eq_(N). The C-bound H atoms were located geometrically (C—H = 0.95–0.99 Å) and refined as riding atoms. The methyl groups were allowed to rotate, but not to tip, to best fit the electron density. The constraint *U*_iso_(H) = 1.2*U*_eq_(parent) or 1.5*U*_eq_(methyl C) was applied in all cases. The structure was refined as a racemic twin, leading to a refined twin fraction of 0.46 (6).

## Supplementary Material

Crystal structure: contains datablock(s) I. DOI: 10.1107/S205698902600561X/hb8226sup1.cif

Structure factors: contains datablock(s) I. DOI: 10.1107/S205698902600561X/hb8226Isup2.hkl

Supporting information file. DOI: 10.1107/S205698902600561X/hb8226Isup3.cml

CCDC reference: 2557328

Additional supporting information:  crystallographic information; 3D view; checkCIF report

## Figures and Tables

**Table 1:** Hydrogen-bond geometry (Å, °)

*D*—H⋯*A*	*D*—H	H⋯*A*	*D*⋯*A*	*D*—H⋯*A*
N1—H1⋯O41	0.97 (3)	1.87 (5)	2.826 (16)	169 (16)
N11—H11⋯O2	0.98 (3)	1.79 (8)	2.698 (15)	153 (14)
N21—H21⋯O44	0.97 (3)	1.89 (7)	2.812 (15)	157 (15)
N31—H31⋯O22	0.98 (3)	1.89 (11)	2.717 (15)	141 (14)
C7—H7⋯S12^i^	0.95	2.97	3.923 (15)	177
C27—H27⋯S32^i^	0.95	2.94	3.875 (15)	169
C34—H34*A*⋯S33^ii^	0.99	2.97	3.94 (2)	168
C37—H37⋯F16^iii^	0.95	2.27	3.17 (2)	160
C42—H42*A*⋯O22^iv^	0.98	2.53	3.259 (19)	131

Symmetry codes: (i) 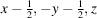; (ii) 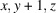; (iii) 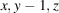; (iv) 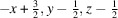.

**Table 2:** Experimental details

Crystal data	
Chemical formula	C_16_H_11_F_2_N_3_OS_2_·C_2_H_6_OS
*M*_r_	441.53
Crystal system, space group	Orthorhombic, *Pna*2_1_
Temperature (K)	100
*a*, *b*, *c* (Å)	18.9344 (11), 4.6861 (3), 44.085 (2)
*V* (Å^3^)	3911.6 (4)
*Z*	8
Radiation type	Cu *K*α
μ (mm^−1^)	3.82
Crystal size (mm)	0.45 × 0.03 × 0.01

Data collection	
Diffractometer	XtaLAB Synergy, Single source at home/near, HyPix-Arc 100
Absorption correction	Multi-scan (*CrysAlis PRO*; Rigaku OD, 2025 ▸)
*T*_min_, *T*_max_	0.521, 1.000
No. of measured, independent and observed [*I* > 2σ(*I*)] reflections	35724, 7168, 5286
*R*_int_	0.146
(sin θ/λ)_max_ (Å^−1^)	0.622

Refinement	
*R*[*F*^2^ > 2σ(*F*^2^)], *wR*(*F*^2^), *S*	0.107, 0.323, 1.23
No. of reflections	7168
No. of parameters	522
No. of restraints	5
H-atom treatment	H atoms treated by a mixture of independent and constrained refinement
Δρ_max_, Δρ_min_ (e Å^−3^)	1.30, −0.89
Absolute structure	Refined as an inversion twin.
Absolute structure parameter	0.46 (6)

Computer programs: *CrysAlis PRO* (Rigaku OD, 2025 ▸), *SHELXT2018/2* (Sheldrick, 2015*a* ▸), *SHELXL2025/1* (Sheldrick, 2015*b* ▸), *Mercury* (Macrae *et al.*, 2020 ▸), *enCIFer* (Allen *et al.*, 2004 ▸), *publCIF* (Westrip, 2010 ▸) and *OLEX2* (Dolomanov *et al.*, 2009 ▸).

## supplementary crystallographic information

### (Z)-2-Fluorobenzyl 2-(5-fluoro-2-oxoindolin-3-ylidene)hydrazinecarbodithioate dimethyl sulfoxide monosolvate Crystal data

**Table:**

C_16_H_11_F_2_N_3_OS_2_·C_2_H_6_OS	*D*_x_ = 1.499 Mg m^−^^3^
*M**_r_* = 441.53	Cu *K*α radiation, λ = 1.54184 Å
Orthorhombic, *P**n**a*2_1_	Cell parameters from 4927 reflections
*a* = 18.9344 (11) Å	θ = 2.0–72.7°
*b* = 4.6861 (3) Å	µ = 3.82 mm^−^^1^
*c* = 44.085 (2) Å	*T* = 100 K
*V* = 3911.6 (4) Å^3^	Needle, yellow
*Z* = 8	0.45 × 0.03 × 0.01 mm
*F*(000) = 1824	

### (Z)-2-Fluorobenzyl 2-(5-fluoro-2-oxoindolin-3-ylidene)hydrazinecarbodithioate dimethyl sulfoxide monosolvate Data collection

**Table:**

XtaLAB Synergy, Single source at home/near, HyPix-Arc 100 diffractometer	7168 independent reflections
Radiation source: micro-focus sealed X-ray tube, PhotonJet (Cu) X-ray Source	5286 reflections with *I* > 2σ(*I*)
Mirror monochromator	*R*_int_ = 0.146
Detector resolution: 10.0000 pixels mm^-1^	θ_max_ = 73.6°, θ_min_ = 2.0°
ω scans	*h* = −21→23
Absorption correction: multi-scan (CrysAlisPro; Rigaku OD, 2025)	*k* = −5→5
*T*_min_ = 0.521, *T*_max_ = 1.000	*l* = −53→53
35724 measured reflections	

### (Z)-2-Fluorobenzyl 2-(5-fluoro-2-oxoindolin-3-ylidene)hydrazinecarbodithioate dimethyl sulfoxide monosolvate Refinement

**Table:**

Refinement on *F*^2^	Hydrogen site location: mixed
Least-squares matrix: full	H atoms treated by a mixture of independent and constrained refinement
*R*[*F*^2^ > 2σ(*F*^2^)] = 0.107	*w* = 1/[σ^2^(*F*_o_^2^) + (0.2*P*)^2^] where *P* = (*F*_o_^2^ + 2*F*_c_^2^)/3
*wR*(*F*^2^) = 0.323	(Δ/σ)_max_ = 0.001
*S* = 1.23	Δρ_max_ = 1.30 e Å^−^^3^
7168 reflections	Δρ_min_ = −0.89 e Å^−^^3^
522 parameters	Absolute structure: Refined as an inversion twin.
5 restraints	Absolute structure parameter: 0.46 (6)
Primary atom site location: dual	

### (Z)-2-Fluorobenzyl 2-(5-fluoro-2-oxoindolin-3-ylidene)hydrazinecarbodithioate dimethyl sulfoxide monosolvate Special details

**Table:**

Geometry. All esds (except the esd in the dihedral angle between two l.s. planes) are estimated using the full covariance matrix. The cell esds are taken into account individually in the estimation of esds in distances, angles and torsion angles; correlations between esds in cell parameters are only used when they are defined by crystal symmetry. An approximate (isotropic) treatment of cell esds is used for estimating esds involving l.s. planes.
Refinement. Refined as a 2-component inversion twin.N-H hydrogens were located from the difference Fourier map and refined isotropically subject to a distance restraint.The fine needle-shaped crystals showed some signs of polycrystallinity, which was reflected in the weak high-angle data and visible smearing of reflections. Attempts were made unsucessfully to identify specific twin laws and process the data to account for these, but all of these led to poorer data metrics. The data presented is the best available, despite the elevated values of R_int_, R_1_, and wR_2_.

### (Z)-2-Fluorobenzyl 2-(5-fluoro-2-oxoindolin-3-ylidene)hydrazinecarbodithioate dimethyl sulfoxide monosolvate Fractional atomic coordinates and isotropic or equivalent isotropic displacement parameters (Å^2^)

**Table:**

	*x*	*y*	*z*	*U*_iso_*/*U*_eq_	
S12	0.97854 (19)	0.6889 (9)	0.36392 (8)	0.0479 (9)	
S13	0.9103 (2)	0.3993 (9)	0.41830 (8)	0.0480 (9)	
S32	0.74225 (19)	0.6890 (9)	0.63560 (8)	0.0473 (8)	
S33	0.6675 (2)	0.4217 (9)	0.58166 (8)	0.0519 (9)	
S41	0.72757 (18)	−0.7669 (8)	0.24866 (8)	0.0461 (8)	
S44	0.49139 (18)	−0.7703 (8)	0.75110 (8)	0.0459 (8)	
F6	0.6174 (5)	−0.575 (2)	0.41317 (17)	0.056 (2)	
F16	0.9006 (6)	0.866 (2)	0.4783 (3)	0.080 (3)	
F26	0.3939 (5)	−0.621 (2)	0.5851 (2)	0.062 (2)	
F36	0.8164 (6)	0.228 (2)	0.5368 (2)	0.070 (3)	
O2	0.8390 (5)	0.138 (2)	0.3059 (2)	0.050 (2)	
O22	0.6022 (5)	0.127 (2)	0.6947 (2)	0.048 (2)	
O41	0.7287 (6)	−0.440 (2)	0.2519 (2)	0.049 (2)	
O44	0.4921 (6)	−0.446 (2)	0.7477 (2)	0.053 (2)	
N1	0.7566 (6)	−0.232 (3)	0.3109 (3)	0.045 (3)	
H1	0.750 (8)	−0.28 (4)	0.2897 (13)	0.055*	
N10	0.8252 (6)	0.145 (3)	0.3743 (3)	0.044 (3)	
N11	0.8737 (6)	0.324 (3)	0.3619 (2)	0.042 (3)	
H11	0.868 (8)	0.31 (4)	0.3398 (8)	0.050*	
N21	0.5200 (6)	−0.240 (3)	0.6890 (2)	0.041 (3)	
H21	0.512 (8)	−0.26 (4)	0.7107 (9)	0.049*	
N30	0.5924 (6)	0.132 (3)	0.6260 (2)	0.042 (3)	
N31	0.6400 (6)	0.308 (3)	0.6385 (3)	0.047 (3)	
H31	0.641 (8)	0.31 (4)	0.6607 (7)	0.056*	
C2	0.8009 (8)	−0.024 (3)	0.3213 (3)	0.045 (3)	
C3	0.7907 (8)	−0.015 (3)	0.3553 (3)	0.044 (3)	
C4	0.7362 (7)	−0.216 (3)	0.3619 (3)	0.044 (3)	
C5	0.7044 (8)	−0.291 (3)	0.3894 (3)	0.046 (3)	
H5	0.718396	−0.209186	0.408180	0.055*	
C6	0.6520 (8)	−0.490 (4)	0.3873 (3)	0.048 (3)	
C7	0.6322 (7)	−0.628 (3)	0.3611 (3)	0.043 (3)	
H7	0.596079	−0.768556	0.361488	0.052*	
C8	0.6666 (8)	−0.558 (3)	0.3336 (3)	0.048 (3)	
H8	0.654717	−0.648351	0.314963	0.058*	
C9	0.7184 (8)	−0.351 (3)	0.3353 (3)	0.045 (3)	
C12	0.9203 (7)	0.468 (3)	0.3798 (3)	0.039 (3)	
C14	0.9831 (8)	0.603 (4)	0.4340 (3)	0.050 (4)	
H14A	1.027816	0.551888	0.423714	0.060*	
H14B	0.974724	0.809779	0.431346	0.060*	
C15	0.9873 (8)	0.529 (4)	0.4674 (3)	0.051 (4)	
C16	0.9477 (9)	0.665 (4)	0.4883 (3)	0.055 (4)	
C17	0.9501 (10)	0.614 (4)	0.5201 (4)	0.062 (4)	
H17	0.921829	0.718732	0.533972	0.074*	
C18	0.9954 (11)	0.406 (4)	0.5294 (4)	0.066 (5)	
H18	0.996661	0.355306	0.550261	0.080*	
C19	1.0397 (10)	0.265 (4)	0.5093 (4)	0.057 (4)	
H19	1.072830	0.128825	0.516641	0.068*	
C20	1.0355 (9)	0.322 (4)	0.4790 (3)	0.056 (4)	
H20	1.065218	0.221037	0.465305	0.068*	
C22	0.5642 (8)	−0.030 (3)	0.6789 (3)	0.041 (3)	
C23	0.5580 (7)	−0.026 (4)	0.6452 (3)	0.044 (3)	
C24	0.5060 (7)	−0.236 (3)	0.6371 (3)	0.041 (3)	
C25	0.4758 (8)	−0.335 (4)	0.6097 (3)	0.045 (3)	
H25	0.491376	−0.264501	0.590669	0.054*	
C26	0.4239 (9)	−0.534 (4)	0.6116 (3)	0.052 (4)	
C27	0.4014 (7)	−0.670 (4)	0.6386 (3)	0.049 (3)	
H27	0.367075	−0.817849	0.638476	0.059*	
C28	0.4322 (8)	−0.574 (3)	0.6652 (3)	0.044 (3)	
H28	0.418578	−0.656530	0.684053	0.053*	
C29	0.4819 (7)	−0.365 (3)	0.6648 (3)	0.043 (3)	
C32	0.6826 (7)	0.472 (3)	0.6207 (3)	0.037 (3)	
C34	0.7366 (9)	0.645 (4)	0.5647 (3)	0.058 (4)	
H34A	0.727272	0.849217	0.568728	0.069*	
H34B	0.783446	0.594867	0.573070	0.069*	
C35	0.7342 (9)	0.585 (4)	0.5307 (3)	0.055 (4)	
C36	0.7758 (8)	0.379 (4)	0.5181 (4)	0.054 (4)	
C37	0.7785 (9)	0.320 (4)	0.4884 (4)	0.057 (4)	
H37	0.809127	0.176520	0.480669	0.069*	
C38	0.7336 (9)	0.481 (4)	0.4690 (3)	0.055 (4)	
H38	0.733153	0.444659	0.447815	0.066*	
C39	0.6909 (10)	0.689 (4)	0.4811 (4)	0.060 (4)	
H39	0.661042	0.796823	0.468202	0.072*	
C40	0.6911 (10)	0.741 (4)	0.5117 (4)	0.063 (5)	
H40	0.661413	0.885835	0.519875	0.075*	
C42	0.8062 (8)	−0.862 (4)	0.2295 (3)	0.048 (3)	
H42A	0.809978	−0.752360	0.210676	0.072*	
H42B	0.805309	−1.066396	0.224799	0.072*	
H42C	0.846927	−0.820290	0.242516	0.072*	
C43	0.6681 (9)	−0.839 (4)	0.2190 (3)	0.056 (4)	
H43A	0.619786	−0.801379	0.225898	0.084*	
H43B	0.672329	−1.039938	0.213035	0.084*	
H43C	0.679053	−0.716876	0.201626	0.084*	
C45	0.4318 (9)	−0.844 (4)	0.7816 (4)	0.060 (4)	
H45A	0.448086	−0.747030	0.799966	0.090*	
H45B	0.430098	−1.050294	0.785170	0.090*	
H45C	0.384503	−0.775290	0.776239	0.090*	
C46	0.5691 (8)	−0.859 (4)	0.7703 (3)	0.052 (4)	
H46A	0.609942	−0.794757	0.758528	0.078*	
H46B	0.571448	−1.066476	0.773026	0.078*	
H46C	0.569410	−0.765889	0.790234	0.078*	

### (Z)-2-Fluorobenzyl 2-(5-fluoro-2-oxoindolin-3-ylidene)hydrazinecarbodithioate dimethyl sulfoxide monosolvate Atomic displacement parameters (Å^2^)

**Table:**

	*U*^11^	*U*^22^	*U*^33^	*U*^12^	*U*^13^	*U*^23^
S12	0.057 (2)	0.049 (2)	0.0372 (17)	−0.0026 (16)	0.0009 (13)	0.0037 (17)
S13	0.063 (2)	0.051 (2)	0.0296 (13)	−0.0094 (17)	−0.0018 (13)	0.0016 (15)
S32	0.057 (2)	0.0462 (19)	0.0381 (17)	−0.0024 (15)	−0.0019 (14)	−0.0024 (17)
S33	0.067 (2)	0.058 (2)	0.0308 (14)	−0.0131 (18)	0.0017 (14)	0.0008 (16)
S41	0.062 (2)	0.050 (2)	0.0269 (15)	−0.0080 (16)	−0.0008 (13)	−0.0023 (14)
S44	0.0580 (19)	0.051 (2)	0.0291 (15)	−0.0024 (16)	0.0008 (13)	0.0022 (14)
F6	0.077 (5)	0.061 (6)	0.031 (4)	−0.013 (5)	0.008 (3)	0.019 (4)
F16	0.087 (7)	0.063 (7)	0.089 (8)	0.019 (6)	0.005 (6)	−0.010 (6)
F26	0.063 (5)	0.076 (7)	0.046 (5)	−0.008 (5)	−0.007 (4)	−0.007 (5)
F36	0.098 (7)	0.069 (7)	0.042 (4)	0.010 (6)	−0.004 (4)	0.012 (5)
O2	0.064 (6)	0.055 (6)	0.031 (5)	0.009 (5)	0.006 (4)	0.009 (5)
O22	0.064 (6)	0.052 (6)	0.028 (4)	−0.015 (5)	−0.007 (4)	−0.002 (4)
O41	0.076 (7)	0.037 (5)	0.035 (5)	−0.001 (4)	0.000 (4)	0.004 (5)
O44	0.072 (7)	0.049 (6)	0.040 (5)	0.000 (5)	−0.005 (5)	0.005 (5)
N1	0.051 (7)	0.058 (9)	0.027 (6)	−0.003 (6)	0.003 (4)	0.004 (5)
N10	0.052 (7)	0.042 (7)	0.037 (6)	0.006 (5)	−0.002 (4)	0.005 (5)
N11	0.049 (6)	0.045 (6)	0.031 (5)	−0.019 (5)	−0.006 (4)	0.003 (5)
N21	0.057 (7)	0.043 (7)	0.023 (5)	0.000 (5)	−0.001 (4)	−0.002 (5)
N30	0.052 (7)	0.047 (7)	0.028 (5)	0.004 (5)	0.001 (4)	0.001 (5)
N31	0.054 (7)	0.054 (7)	0.032 (5)	−0.007 (5)	0.008 (5)	0.002 (6)
C2	0.067 (9)	0.040 (8)	0.027 (6)	0.008 (7)	0.006 (5)	0.018 (6)
C3	0.062 (8)	0.044 (8)	0.026 (6)	0.000 (7)	−0.001 (5)	0.003 (6)
C4	0.043 (7)	0.050 (9)	0.040 (7)	0.001 (6)	−0.005 (5)	−0.007 (7)
C5	0.067 (9)	0.043 (8)	0.028 (6)	0.016 (7)	0.000 (6)	0.011 (6)
C6	0.057 (8)	0.058 (10)	0.031 (6)	0.005 (7)	0.003 (5)	0.016 (7)
C7	0.052 (7)	0.040 (8)	0.038 (7)	−0.005 (6)	0.003 (5)	0.009 (6)
C8	0.062 (9)	0.051 (9)	0.032 (6)	0.004 (7)	0.005 (6)	−0.005 (6)
C9	0.057 (8)	0.059 (10)	0.020 (5)	0.011 (7)	0.009 (5)	0.010 (6)
C12	0.042 (7)	0.042 (8)	0.034 (6)	0.002 (6)	0.001 (5)	0.005 (6)
C14	0.053 (9)	0.047 (9)	0.051 (8)	−0.003 (7)	−0.010 (6)	0.005 (7)
C15	0.064 (9)	0.042 (8)	0.048 (8)	−0.011 (7)	0.000 (6)	−0.013 (7)
C16	0.077 (10)	0.055 (10)	0.034 (7)	−0.001 (8)	−0.003 (6)	−0.001 (7)
C17	0.081 (11)	0.055 (11)	0.049 (9)	−0.007 (9)	0.005 (7)	−0.010 (8)
C18	0.101 (13)	0.063 (12)	0.035 (7)	−0.011 (11)	0.002 (8)	−0.008 (8)
C19	0.085 (11)	0.037 (9)	0.050 (8)	0.001 (7)	−0.009 (8)	−0.010 (7)
C20	0.065 (9)	0.070 (11)	0.035 (7)	−0.017 (8)	−0.001 (6)	−0.005 (7)
C22	0.061 (8)	0.033 (7)	0.031 (6)	0.007 (6)	0.005 (5)	0.005 (6)
C23	0.053 (8)	0.051 (9)	0.027 (5)	−0.001 (6)	−0.006 (5)	−0.003 (6)
C24	0.059 (7)	0.043 (8)	0.020 (6)	0.008 (6)	0.003 (5)	0.001 (5)
C25	0.050 (8)	0.057 (9)	0.028 (6)	0.000 (7)	−0.002 (5)	−0.003 (6)
C26	0.064 (9)	0.067 (11)	0.024 (5)	0.004 (8)	−0.007 (5)	0.000 (6)
C27	0.043 (7)	0.053 (9)	0.051 (8)	−0.015 (6)	0.006 (6)	−0.001 (7)
C28	0.063 (8)	0.044 (8)	0.027 (6)	0.007 (6)	0.002 (5)	0.000 (6)
C29	0.047 (7)	0.050 (9)	0.032 (6)	−0.004 (6)	−0.004 (5)	−0.002 (6)
C32	0.059 (8)	0.027 (6)	0.024 (5)	0.002 (6)	0.001 (5)	0.003 (5)
C34	0.077 (11)	0.061 (11)	0.034 (7)	−0.014 (8)	0.004 (6)	0.002 (7)
C35	0.065 (9)	0.065 (12)	0.034 (7)	−0.016 (8)	−0.002 (6)	0.006 (7)
C36	0.060 (9)	0.053 (10)	0.048 (8)	−0.009 (7)	−0.005 (6)	0.023 (8)
C37	0.080 (11)	0.053 (10)	0.039 (7)	0.003 (8)	0.006 (7)	0.006 (7)
C38	0.076 (10)	0.049 (9)	0.040 (7)	0.007 (8)	0.004 (6)	0.010 (7)
C39	0.074 (11)	0.059 (11)	0.048 (8)	−0.003 (8)	−0.003 (7)	0.011 (8)
C40	0.077 (12)	0.058 (11)	0.053 (9)	0.000 (9)	−0.005 (8)	0.001 (8)
C42	0.055 (8)	0.050 (9)	0.039 (7)	0.000 (7)	−0.001 (5)	−0.001 (6)
C43	0.065 (10)	0.065 (11)	0.038 (7)	0.004 (8)	−0.007 (6)	0.013 (7)
C45	0.065 (10)	0.068 (11)	0.046 (8)	0.009 (8)	0.000 (6)	0.003 (8)
C46	0.057 (9)	0.066 (10)	0.034 (6)	−0.017 (7)	−0.001 (5)	−0.018 (7)

### (Z)-2-Fluorobenzyl 2-(5-fluoro-2-oxoindolin-3-ylidene)hydrazinecarbodithioate dimethyl sulfoxide monosolvate Geometric parameters (Å, º)

**Table:**

S12—C12	1.665 (14)	C15—C16	1.35 (2)
S13—C12	1.739 (13)	C15—C20	1.43 (3)
S13—C14	1.814 (15)	C16—C17	1.42 (2)
S32—C32	1.658 (14)	C17—H17	0.9500
S33—C32	1.758 (12)	C17—C18	1.36 (3)
S33—C34	1.834 (17)	C18—H18	0.9500
S41—O41	1.536 (11)	C18—C19	1.39 (3)
S41—C42	1.768 (15)	C19—H19	0.9500
S41—C43	1.758 (15)	C19—C20	1.37 (2)
S44—O44	1.525 (12)	C20—H20	0.9500
S44—C45	1.788 (17)	C22—C23	1.490 (15)
S44—C46	1.748 (16)	C23—C24	1.44 (2)
F6—C6	1.373 (14)	C24—C25	1.413 (18)
F16—C16	1.37 (2)	C24—C29	1.434 (18)
F26—C26	1.361 (16)	C25—H25	0.9500
F36—C36	1.332 (17)	C25—C26	1.36 (2)
O2—C2	1.250 (17)	C26—C27	1.42 (2)
O22—C22	1.244 (17)	C27—H27	0.9500
N1—H1	0.97 (3)	C27—C28	1.39 (2)
N1—C2	1.37 (2)	C28—H28	0.9500
N1—C9	1.413 (17)	C28—C29	1.36 (2)
N10—N11	1.359 (17)	C34—H34A	0.9900
N10—C3	1.301 (19)	C34—H34B	0.9900
N11—H11	0.98 (3)	C34—C35	1.52 (2)
N11—C12	1.364 (17)	C35—C36	1.36 (3)
N21—H21	0.97 (3)	C35—C40	1.38 (2)
N21—C22	1.369 (19)	C36—C37	1.34 (2)
N21—C29	1.415 (17)	C37—H37	0.9500
N30—N31	1.341 (17)	C37—C38	1.42 (2)
N30—C23	1.300 (19)	C38—H38	0.9500
N31—H31	0.98 (3)	C38—C39	1.37 (3)
N31—C32	1.361 (17)	C39—H39	0.9500
C2—C3	1.509 (16)	C39—C40	1.37 (2)
C3—C4	1.42 (2)	C40—H40	0.9500
C4—C5	1.399 (19)	C42—H42A	0.9800
C4—C9	1.37 (2)	C42—H42B	0.9800
C5—H5	0.9500	C42—H42C	0.9800
C5—C6	1.37 (2)	C43—H43A	0.9800
C6—C7	1.38 (2)	C43—H43B	0.9800
C7—H7	0.9500	C43—H43C	0.9800
C7—C8	1.415 (18)	C45—H45A	0.9800
C8—H8	0.9500	C45—H45B	0.9800
C8—C9	1.38 (2)	C45—H45C	0.9800
C14—H14A	0.9900	C46—H46A	0.9800
C14—H14B	0.9900	C46—H46B	0.9800
C14—C15	1.51 (2)	C46—H46C	0.9800
			
C12—S13—C14	101.1 (7)	O22—C22—C23	126.9 (13)
C32—S33—C34	102.0 (7)	N21—C22—C23	106.5 (12)
O41—S41—C42	106.5 (7)	N30—C23—C22	127.9 (13)
O41—S41—C43	105.7 (7)	N30—C23—C24	125.0 (12)
C43—S41—C42	97.8 (7)	C24—C23—C22	107.1 (12)
O44—S44—C45	105.8 (8)	C25—C24—C23	135.4 (13)
O44—S44—C46	106.1 (7)	C25—C24—C29	117.4 (13)
C46—S44—C45	96.9 (7)	C29—C24—C23	107.1 (11)
C2—N1—H1	125 (10)	C24—C25—H25	121.1
C2—N1—C9	109.7 (11)	C26—C25—C24	117.8 (13)
C9—N1—H1	125 (10)	C26—C25—H25	121.1
C3—N10—N11	115.8 (11)	F26—C26—C27	117.5 (15)
N10—N11—H11	107 (10)	C25—C26—F26	117.1 (12)
N10—N11—C12	120.8 (11)	C25—C26—C27	125.3 (13)
C12—N11—H11	133 (10)	C26—C27—H27	121.9
C22—N21—H21	119 (10)	C28—C27—C26	116.2 (14)
C22—N21—C29	111.3 (11)	C28—C27—H27	121.9
C29—N21—H21	129 (10)	C27—C28—H28	119.6
C23—N30—N31	115.0 (11)	C29—C28—C27	120.8 (13)
N30—N31—H31	116 (10)	C29—C28—H28	119.6
N30—N31—C32	120.5 (11)	N21—C29—C24	107.9 (12)
C32—N31—H31	123 (10)	C28—C29—N21	129.7 (13)
O2—C2—N1	127.2 (12)	C28—C29—C24	122.4 (13)
O2—C2—C3	126.7 (14)	S32—C32—S33	125.5 (8)
N1—C2—C3	106.0 (11)	N31—C32—S32	121.3 (10)
N10—C3—C2	126.2 (13)	N31—C32—S33	113.2 (10)
N10—C3—C4	127.7 (12)	S33—C34—H34A	110.5
C4—C3—C2	106.1 (12)	S33—C34—H34B	110.5
C5—C4—C3	130.9 (14)	H34A—C34—H34B	108.7
C9—C4—C3	107.9 (13)	C35—C34—S33	106.0 (11)
C9—C4—C5	121.2 (15)	C35—C34—H34A	110.5
C4—C5—H5	122.4	C35—C34—H34B	110.5
C6—C5—C4	115.3 (13)	C36—C35—C34	120.9 (15)
C6—C5—H5	122.4	C36—C35—C40	118.1 (15)
F6—C6—C7	115.5 (14)	C40—C35—C34	121.0 (17)
C5—C6—F6	119.4 (12)	F36—C36—C35	117.3 (15)
C5—C6—C7	125.0 (12)	F36—C36—C37	118.1 (16)
C6—C7—H7	120.4	C37—C36—C35	124.6 (15)
C6—C7—C8	119.1 (14)	C36—C37—H37	121.5
C8—C7—H7	120.4	C36—C37—C38	117.0 (16)
C7—C8—H8	121.9	C38—C37—H37	121.5
C9—C8—C7	116.1 (12)	C37—C38—H38	120.2
C9—C8—H8	121.9	C39—C38—C37	119.6 (15)
C4—C9—N1	110.1 (14)	C39—C38—H38	120.2
C4—C9—C8	123.1 (12)	C38—C39—H39	119.7
C8—C9—N1	126.6 (12)	C40—C39—C38	120.6 (17)
S12—C12—S13	126.7 (8)	C40—C39—H39	119.7
N11—C12—S12	119.6 (9)	C35—C40—H40	120.0
N11—C12—S13	113.6 (10)	C39—C40—C35	120.1 (18)
S13—C14—H14A	110.3	C39—C40—H40	120.0
S13—C14—H14B	110.3	S41—C42—H42A	109.5
H14A—C14—H14B	108.6	S41—C42—H42B	109.5
C15—C14—S13	107.0 (11)	S41—C42—H42C	109.5
C15—C14—H14A	110.3	H42A—C42—H42B	109.5
C15—C14—H14B	110.3	H42A—C42—H42C	109.5
C16—C15—C14	121.8 (16)	H42B—C42—H42C	109.5
C16—C15—C20	115.8 (15)	S41—C43—H43A	109.5
C20—C15—C14	122.3 (14)	S41—C43—H43B	109.5
F16—C16—C17	117.2 (15)	S41—C43—H43C	109.5
C15—C16—F16	117.7 (14)	H43A—C43—H43B	109.5
C15—C16—C17	125.1 (17)	H43A—C43—H43C	109.5
C16—C17—H17	122.0	H43B—C43—H43C	109.5
C18—C17—C16	116.0 (16)	S44—C45—H45A	109.5
C18—C17—H17	122.0	S44—C45—H45B	109.5
C17—C18—H18	119.0	S44—C45—H45C	109.5
C17—C18—C19	122.0 (16)	H45A—C45—H45B	109.5
C19—C18—H18	119.0	H45A—C45—H45C	109.5
C18—C19—H19	120.1	H45B—C45—H45C	109.5
C20—C19—C18	119.8 (18)	S44—C46—H46A	109.5
C20—C19—H19	120.1	S44—C46—H46B	109.5
C15—C20—H20	119.4	S44—C46—H46C	109.5
C19—C20—C15	121.3 (16)	H46A—C46—H46B	109.5
C19—C20—H20	119.4	H46A—C46—H46C	109.5
O22—C22—N21	126.7 (11)	H46B—C46—H46C	109.5
			
S13—C14—C15—C16	−85.2 (18)	C9—C4—C5—C6	−5 (2)
S13—C14—C15—C20	97.6 (15)	C12—S13—C14—C15	−172.2 (11)
S33—C34—C35—C36	−92.3 (16)	C14—S13—C12—S12	−4.7 (12)
S33—C34—C35—C40	89.1 (18)	C14—S13—C12—N11	176.2 (11)
F6—C6—C7—C8	−178.4 (12)	C14—C15—C16—F16	3 (2)
F16—C16—C17—C18	177.5 (15)	C14—C15—C16—C17	−178.2 (16)
F26—C26—C27—C28	−179.2 (13)	C14—C15—C20—C19	178.2 (16)
F36—C36—C37—C38	178.9 (15)	C15—C16—C17—C18	−1 (3)
O2—C2—C3—N10	−7 (2)	C16—C15—C20—C19	1 (2)
O2—C2—C3—C4	172.6 (14)	C16—C17—C18—C19	4 (3)
O22—C22—C23—N30	3 (3)	C17—C18—C19—C20	−4 (3)
O22—C22—C23—C24	−177.7 (14)	C18—C19—C20—C15	1 (3)
N1—C2—C3—N10	176.7 (15)	C20—C15—C16—F16	−179.8 (14)
N1—C2—C3—C4	−3.4 (15)	C20—C15—C16—C17	−1 (3)
N10—N11—C12—S12	−179.3 (10)	C22—N21—C29—C24	3.3 (15)
N10—N11—C12—S13	−0.2 (18)	C22—N21—C29—C28	−178.7 (14)
N10—C3—C4—C5	1 (3)	C22—C23—C24—C25	179.4 (16)
N10—C3—C4—C9	−175.8 (15)	C22—C23—C24—C29	−0.5 (15)
N11—N10—C3—C2	2 (2)	C23—N30—N31—C32	175.8 (13)
N11—N10—C3—C4	−178.3 (14)	C23—C24—C25—C26	−176.8 (16)
N21—C22—C23—N30	−177.2 (14)	C23—C24—C29—N21	−1.6 (15)
N21—C22—C23—C24	2.4 (15)	C23—C24—C29—C28	−179.8 (13)
N30—N31—C32—S32	−179.4 (10)	C24—C25—C26—F26	178.1 (13)
N30—N31—C32—S33	0.0 (17)	C24—C25—C26—C27	−6 (3)
N30—C23—C24—C25	−1 (3)	C25—C24—C29—N21	178.5 (12)
N30—C23—C24—C29	179.1 (14)	C25—C24—C29—C28	0 (2)
N31—N30—C23—C22	1 (2)	C25—C26—C27—C28	5 (2)
N31—N30—C23—C24	−178.2 (13)	C26—C27—C28—C29	−1 (2)
C2—N1—C9—C4	1.5 (17)	C27—C28—C29—N21	−179.2 (14)
C2—N1—C9—C8	177.3 (14)	C27—C28—C29—C24	−1 (2)
C2—C3—C4—C5	−178.6 (15)	C29—N21—C22—O22	176.6 (14)
C2—C3—C4—C9	4.3 (16)	C29—N21—C22—C23	−3.5 (15)
C3—N10—N11—C12	−169.8 (13)	C29—C24—C25—C26	3 (2)
C3—C4—C5—C6	178.7 (15)	C32—S33—C34—C35	172.7 (12)
C3—C4—C9—N1	−3.7 (17)	C34—S33—C32—S32	3.7 (12)
C3—C4—C9—C8	−179.7 (14)	C34—S33—C32—N31	−175.7 (11)
C4—C5—C6—F6	−179.5 (13)	C34—C35—C36—F36	2 (2)
C4—C5—C6—C7	4 (2)	C34—C35—C36—C37	−177.7 (17)
C5—C4—C9—N1	178.9 (13)	C34—C35—C40—C39	178.5 (16)
C5—C4—C9—C8	3 (2)	C35—C36—C37—C38	−1 (3)
C5—C6—C7—C8	−2 (2)	C36—C35—C40—C39	0 (3)
C6—C7—C8—C9	0 (2)	C36—C37—C38—C39	1 (3)
C7—C8—C9—N1	−175.7 (14)	C37—C38—C39—C40	0 (3)
C7—C8—C9—C4	0 (2)	C38—C39—C40—C35	0 (3)
C9—N1—C2—O2	−174.8 (14)	C40—C35—C36—F36	−179.2 (15)
C9—N1—C2—C3	1.2 (16)	C40—C35—C36—C37	1 (3)

### (Z)-2-Fluorobenzyl 2-(5-fluoro-2-oxoindolin-3-ylidene)hydrazinecarbodithioate dimethyl sulfoxide monosolvate Hydrogen-bond geometry (Å, º)

**Table:**

*D*—H···*A*	*D*—H	H···*A*	*D*···*A*	*D*—H···*A*
N1—H1···O41	0.97 (3)	1.87 (5)	2.826 (16)	169 (16)
N11—H11···O2	0.98 (3)	1.79 (8)	2.698 (15)	153 (14)
N21—H21···O44	0.97 (3)	1.89 (7)	2.812 (15)	157 (15)
N31—H31···O22	0.98 (3)	1.89 (11)	2.717 (15)	141 (14)
C7—H7···S12^i^	0.95	2.97	3.923 (15)	177
C27—H27···S32^i^	0.95	2.94	3.875 (15)	169
C34—H34*A*···S33^ii^	0.99	2.97	3.94 (2)	168
C37—H37···F16^iii^	0.95	2.27	3.17 (2)	160
C42—H42*A*···O22^iv^	0.98	2.53	3.259 (19)	131

Symmetry codes: (i) *x*−1/2, −*y*−1/2, *z*; (ii) *x*, *y*+1, *z*; (iii) *x*, *y*−1, *z*; (iv) −*x*+3/2, *y*−1/2, *z*−1/2.
